# Mid-long-term results of total knee arthroplasty followed by ipsilateral total hip arthroplasty versus total hip arthroplasty subsequent to ipsilateral total knee arthroplasty: a case-control analysis

**DOI:** 10.1186/s12891-021-04455-7

**Published:** 2021-06-24

**Authors:** Zunhan Liu, Wei-Nan Zeng, Zhenyu Luo, Enze Zhao, Hao Li, Zongke Zhou

**Affiliations:** Department of Orthopedic Surgery, West China Hospital, West China Medical School, Sichuan University, #37 Guoxue Road, Chengdu, Sichuan Province 610041 People’s Republic of China

**Keywords:** Total knee arthroplasty, Total hip arthroplasty, Ipsilateral, Mechanical axis, Valgus

## Abstract

**Background:**

The aim of the present study was to compare the outcomes of patients who underwent different sequences of ipsilateral total hip arthroplasty (THA) and total knee arthroplasty (TKA).

**Methods:**

We retrospectively identified 47 patients who underwent TKA followed by ipsilateral THA (THA-TKA) and 36 patients who received THA subsequent to ipsilateral TKA (TKA-THA) for rheumatoid arthritis or osteoarthritis between January 2008 and April 2014. Twenty-eight patients were selected for each group after case-control matching with preoperative demographics and protheses of THA. Clinical scores, radiographic results, complication rates, and survivorship were compared. The median duration of follow-up was 110 (range 80–149) months.

**Results:**

Both groups showed significant improvement in Harris Hip Scores, Knee Society Score, and Short Form-12 at the last follow-up compared to baseline (*p* < .001). At the last follow-up, all clinical scores were actually lower in the THA-TKA group, but those differences were not statistically significant. Otherwise, there was no significant difference in radiological alignment or complication rates. The survivorship of THA and TKA in the THA-TKA group was 94.7 and 95.7%, respectively, compared with 92.4 and 100.0% in the TKA-THA group at 8 years (log rank, *p* = .939 and .187).

**Conclusions:**

Patients who underwent ipsilateral THA and TKA with different sequences achieved similar favorable outcomes. Total joint arthroplasty can be performed safely with excellent outcomes in patients with a history of prior ipsilateral THA or TKA.

**Trial registration:**

The trial was registered in the Chinese Clinical Trial Registry (ChiCTR2000035147) dated 2 August 2020.

## Background

Although the utilization of disease-modifying medications has improved the quality of life of patients with symptomatic arthritis, the number of people affected with multiple lower extremity joints remains high [1, 2]. Subsequently, the possibility of performing total joint arthroplasty (TJA) on the ipsilateral hip and knee in the same patient may increase. A previous study reported that the cumulative incidences of total knee arthroplasty (TKA) followed by ipsilateral total hip arthroplasty (THA) and THA subsequent to ipsilateral TKA were 2.1 and 1.8%, respectively, at the 20-year follow-up [3].

TJA is one of the most successful health care interventions. Patients with end-stage degenerative or inflammatory arthritis report excellent outcomes after THA or TKA [4]. However, there is some evidence to suggest loading redistribution and biomechanical changes in the lower limb after THA or TKA [5–9]. Data concerning the influence of prior THA on the ipsilateral knee joint or ipsilateral TKA remain controversial. Several studies have reported that the lateral patellar tilt increased and the external knee adduction moment decreased after ipsilateral THA, which may lead to abnormal loading on the knees and ipsilateral knee pain [5, 7, 8]. Other studies have suggested that there is no increase in biomechanical loading during gait and posture on ipsilateral or contralateral knee joints after unilateral THA [10]. Furthermore, in certain cases, it is difficult to thoroughly insert the femoral intramedullary guide into the femoral canal during ipsilateral TKA due to a prior femoral prosthesis, increasing the chance of malalignment [11]. To date, only one study has analyzed the influence of prior THA on the functional outcome of subsequent ipsilateral TKA, which demonstrated the influence is limited [12]. On the other hand, the influence of a prior TKA on the ipsilateral hip has also been examined. Previous studies have reported that prior TKA leads to biomechanical changes and that knee alignment caused by prior TKA affects the positioning of the ipsilateral femoral component during THA [6, 13–15]. However, to our knowledge, no study has yet compared the clinical outcomes and implant survivorships in patients undergoing different sequences of ipsilateral THA and TKA.

Therefore, the primary purpose of this study was to determine whether the different sequences of ipsilateral THA and TKA would have different clinical scores. As a secondary outcome, we examined whether the different sequences of ipsilateral THA and TKA would lead to different radiological alignments or component positions, complication rates, and implant survivorships. We hypothesized that patients undergoing different sequences of ipsilateral THA and TKA would have similar clinical outcomes, complication rates, and implant survivorship at the last follow-up.

## Methods

### Patients

This retrospective study was approved by the Institutional Review Board of West China Hospital (ID: 2012–268) and was reported in accordance with the STROCSS criteria [16]. The inclusion criteria were patients with severe pain and/or considerable difficulty in performing daily activities refractory to nonoperative management who underwent ipsilateral THA and TKA for rheumatoid arthritis (RA) or osteoarthritis (OA). The exclusion criteria were hip dysplasia, acetabulum or femoral fracture, ankylosing spondylitis, prior lower extremity fracture, posttraumatic arthritis, revision THA or TKA, and incomplete clinical or radiographic records. Additionally, to avoid bias related to prior TJA, patients with an interval time between ipsilateral THA and TKA shorter than 6 months and patients who underwent prior TJA with a functional score at the time of subsequent surgery less than 70 points were also excluded. We identified 88 patients (97 hips and 97 knees) who underwent ipsilateral THA and TKA at our institution between January 2008 and April 2014. Of these, two patients (2 hips and 2 knees) were lost to follow-up and could not be contacted via telephone, while three patients (4 hips and 4 knees) died from diseases unrelated to the operation. According to different sequences of ipsilateral THA and TKA, the remaining 83 patients (91 hips and 91 knees), including 47 patients undergoing TKA followed by ipsilateral THA and 36 as THA subsequent to ipsilateral TKA, were classified into 2 groups. To minimize possible confounding factors, the two groups were statistically matched for age (up to ±10 years), sex, cause of TJA, primary surgery data (up to ±12 months), and prosthesis of THA by utilizing case-control matching at a 1:1 ratio. Because all implants of primary TKA in our center were posterior-stabilized, we did not match the prosthesis of TKA. Ultimately, 28 patients were selected for each group and included in the final analysis. Table 1 shows and compares demographic characteristics between the 2 groups. Case-control matching was conducted using SPSS statistical software, version 25.0 (IBM Corp., Armonk, NY).

**Table 1:** Baseline characteristics of the 2 case control groups

Parameters	THA-TKA group	TKA-THA group	*P* value
	*n* = 28	*n* = 28	
Gender (male/female)	3/25	3/25	1.000^c^
Age (yr)	57.3 ± 10.6	58.8 ± 10.2	0.610^a^
Body mass index (kg/m^2^)	22.3 (20.2–24.9)	22.2 (19.4–24.8)	0.928^b^
ASA (I-II/III-IV)	19/9	22/6	0.547^c^
No. of diseases (RA/OA)	17/11	17/11	1.000^c^
Varus/neutral/valgus/severe valgus	2/11/14/1	3/6/17/2	0.591^c^
THA follow-up (mo)	109.0 (100.3 to 120.3)	97.5 (82.3 to119.3)	0.095^b^
TKA follow-up (mo)	100.0 (88.8 to 111.0)	114.5 (94.3 to 134.3)	0.012^b^
Interval between surgery (mo)	11.0 (7.3 to 14.8)	15.0 (8.3 to 19.5)	0.061^b^

*ASA* American Society of Anesthesiologists Scale, *RA* Rheumatoid arthritis, *OA* Osteoarthritis

^a^Student *t* test

^b^Mann-Whitney *U* test

^c^Fisher’s exact test

### Surgical techniques

All THA procedures were performed using a posterolateral approach under general anesthesia. The cementless porous-coat acetabular fixations (Pinnacle implants, DePuy Orthopaedics) were routinely press-fitted into the acetabulum at 15 ± 10° of anteversion and 40 ± 10° of inclination. If necessary, supplemental screws were used to achieve implant stability. Two cementless femoral stems, Corail and Summit (DePuy Synthes, Warsaw, IN), were inserted into the hips. Of these, ceramic-on-ceramic articulations were utilized in 42 hips (75.0%), and ceramic-on-polyethylene was used in 14 hips (25.0%).

For the ipsilateral TKA procedure, the knee was exposed by a standard medial parapatellar approach, and osteophytes, worn meniscus, and posterior cruciate ligament of the knee were resected. After determining the entry point, we inserted a femoral intramedullary alignment rod in the center of the femoral intercondylar notch with the distal femoral cutting guide set for individual degrees measured before surgery. Of note, one knee in the THA-TKA group and 2 knees in the TKA-THA group had a preoperative anatomic valgus of > 20° that we released the iliotibial band (ITB) using the “pie-crusting” technique [17]. If the lateral tension on extension and flexion was still tight, the posterolateral capsule was also released, avoiding the collateral ligament (LCL) and popliteus tendon (POP). After assessing the extension gap and balancing the flexion gap, we performed cemented, posterior-stabilized TKAs on all subjects with two total knee implants (DePuy Sigma PFC and Stryker Scorpio NRG). All patients received intraoperative and postoperative prophylactic broad-spectrum antibiotics and tranexamic acid antithrombotic therapy. Postoperatively, active flexion-extension ankle motion and quadriceps strengthening exercises were encouraged. Partial weight-bearing with crutches as tolerated on the second postoperative day and full weight-bearing were allowed from the third day. For patients with THA, simultaneous flexion and internal rotation were avoided after surgery.

### Clinical evaluations and radiographic assessments

**Fig. 1:**
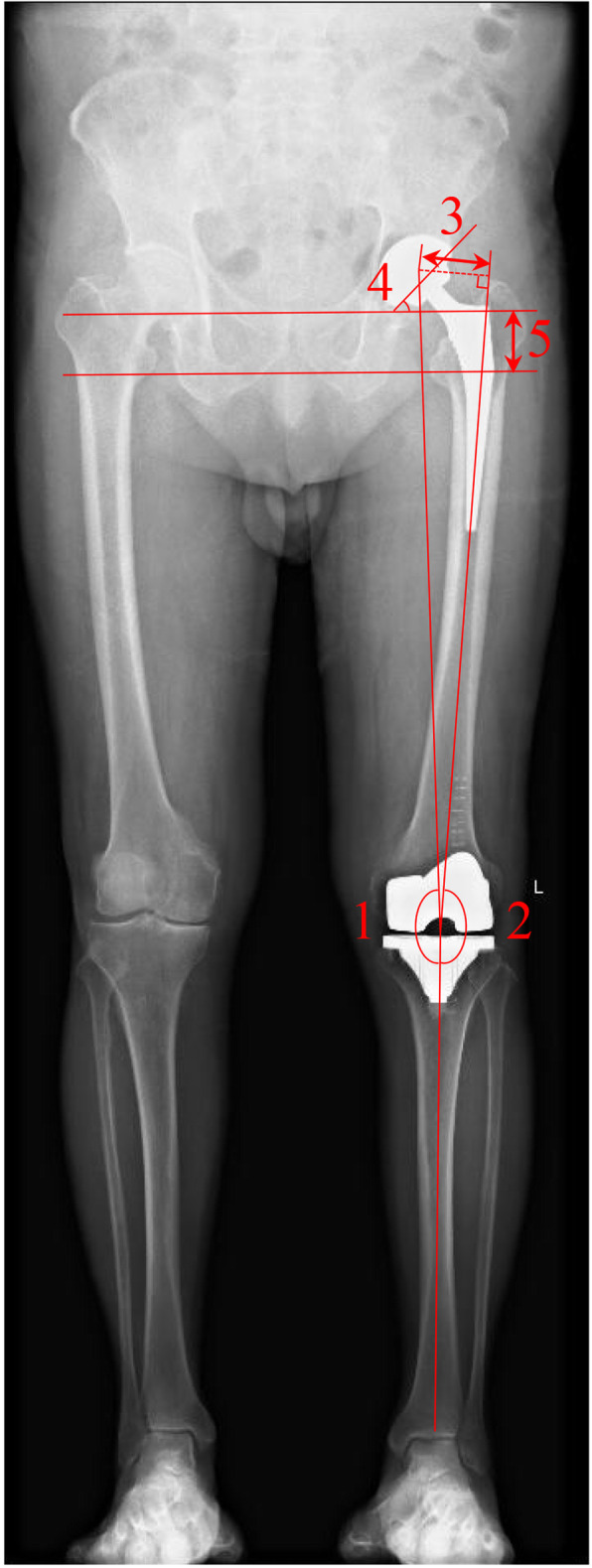
Radiological angles. 1 Hip-knee-ankle angle (HKA). 2 Femorotibial angle (FTA). 3 Femoral offset (FO). 4 Cup inclination angle. 5 Limb length discrepancy (LLD)

Clinical follow-up was conducted routinely at 3 weeks, 3 months and 6 months after the procedures and annually thereafter until the final follow-up. The clinical evaluation protocol included the Harris Hip Score (HHS) [18], Knee Society Score (KSS) [19], and Short Form-12 scale (SF-12) [20]. Radiographs (serial standardized anteroposterior and lateral radiographs of hip and knee, and full-length weight-bearing anteroposterior films) were taken for patients preoperatively and performed at each follow-up. To minimize the variability of the interobserver, all radiographic measurements were performed independently and averaged by 2 trained investigators in the index surgery. To describe the coronal extremity axis, hip-knee-ankle (HKA) angle, femorotibial angle (FTA), femoral offset (FO), inclination angle, and limb length discrepancy (LLD) were measured and recorded. The radiological results are represented by the parameters, as shown in Fig. 1. Of note, when assessing coronal alignment on full-length films, an HKA less than 177° was considered varus, neutral between 177° and 183°, and valgus greater than 183°. Of note, severe valgus knee alignment was defined as an FTA of < 160° [21].

Serial radiographs were also evaluated for evidence of postoperative periprosthetic fracture (PFF), dislocation, subsidence, and femoral component stability. Subsidence of the femoral component was defined as the change in the distance from the center of the femoral head to the lesser trochanter by the method of Heekin et al. [22]. The femoral component stability was evaluated and graded as bone stable, fibrous stable, or unstable, according to the criteria described by Engh et al. [23]. Complications included periprosthetic infection, deep venous thrombosis, and neurologic injury. Kaplan-Meier survivorship analysis was performed on all THAs and TKAs using a standard case scenario where all arthroplasties were considered to be successful at the final follow-up. Prosthesis failure was defined as any reason for aseptic revision.

### Statistical analysis

All statistical analyses were performed using SPSS statistical software, version 25.0 (IBM Corp., Armonk, NY). The figures were generated by GraphPad Prism version 8.0. The normal distribution of the data was tested using Kolmogorov-Smirnov’s test. Depending on the distribution, categorical variables were compared using Fisher’s exact test while continuous variables were evaluated with the student’ t-test for normal distribution data or the Mann-Whitney U-test for skewness distribution data. Means and standard deviation for parametric or medians and 25–75% interquartile ranges for non-parametric data are present. Implant survivorships were analyzed by Kaplan-Meier curves with revision for any reason other than infection as the endpoint. The survival rate between the 2 groups was compared by the log-rank test. α = .05, *P* < .05 indicated statistical significance. A post hoc power analysis was performed to determine the sample size necessary to distinguish differences in HSS and KSS at the follow-up intervals. According to the previous studies [24, 25], a 10-point difference in HSS or KSS was considered clinically significant. An alpha error probability and power were set to .05 and 80% using software (PASS, version 19.0), respectively. The required sample size was 33 patients for each group.

## Results

The preoperative and postoperative clinical scores and radiological results are summarized in Table 2. No significant difference was detected in preoperative clinical parameters, including HHS, KSS, and SF-12. In both groups, the mean HHS, KSS, and SF-12 score significantly improved from preoperatively to the last follow-up (*p* < .001). At the last clinical evaluation, the postoperative parameters, including HHS, KSS-knee score and function score, physical component summary (PCS) and mental component summary (MCS) of SF-12, were actually lower in the THA-TKA group. However, those differences were not significant (*p* > .05).

**Table 2:** Preoperative and postoperative clinical outcomes

Parameters	THA-TKA group	TKA-THA group	*P* value
	*n* = 28	*n* = 28	
Harris Hip Score			
Preoperative	34.0 ± 4.3	34.5 ± 6.0	0.722
Last follow-up	82.2 ± 6.4	83.4 ± 6.9	0.496
Knee Society Score			
Preoperative knee score	32.4 ± 6.4	32.4 ± 8.6	0.986
Last follow-up knee score	86.1 ± 6.3	86.4 ± 8.4	0.901
Preoperative function score	38.0 ± 6.0	37.0 ± 6.0	0.506
Last follow-up function score	85.0 ± 4.3	86.1 ± 4.8	0.382
SF-12			
Preoperative PCS	10.5 ± 2.4	10.6 ± 2.6	0.831
Last follow-up PCS	20.8 ± 2.2	21.0 ± 2.1	0.804
Preoperative MCS	13.3 ± 2.5	13.5 ± 2.9	0.730
Last follow-up MCS	22.9 ± 1.8	23.1 ± 1.9	0.519
HKA (°)			
Preoperative	184.1 ± 5.3	184.1 ± 5.5	0.496
Last follow-up	179.7 ± 3.3	179.6 ± 2.4	0.645
FTA (°)			
Preoperative	173.0 ± 5.5	173.2 ± 5.8	0.600
Last follow-up	177.7 ± 3.2	177.6 ± 2.3	0.178
FO			
Preoperative	39.0 ± 9.0	39.8 ± 7.9	0.705
Last follow-up	40.5 ± 6.5	40.4 ± 9.5	0.967
LLD			
Preoperative	37.8 ± 11.7	41.2 ± 8.1	0.149
Last follow-up	43.0 ± 8.0	46.5 ± 9.1	0.137
Cup inclination (°)	41.9 ± 5.9	40.3 ± 6.2	0.323

The values are present as the mean ± SD

*PCS* Physical component summary, *MCS* Mental component summary, *HKA* Hip knee ankle angle, *FTA* Femorotibial angle, *FO* Femoral offset, *LLD* Limb length discrepancy

**Fig. 2:**
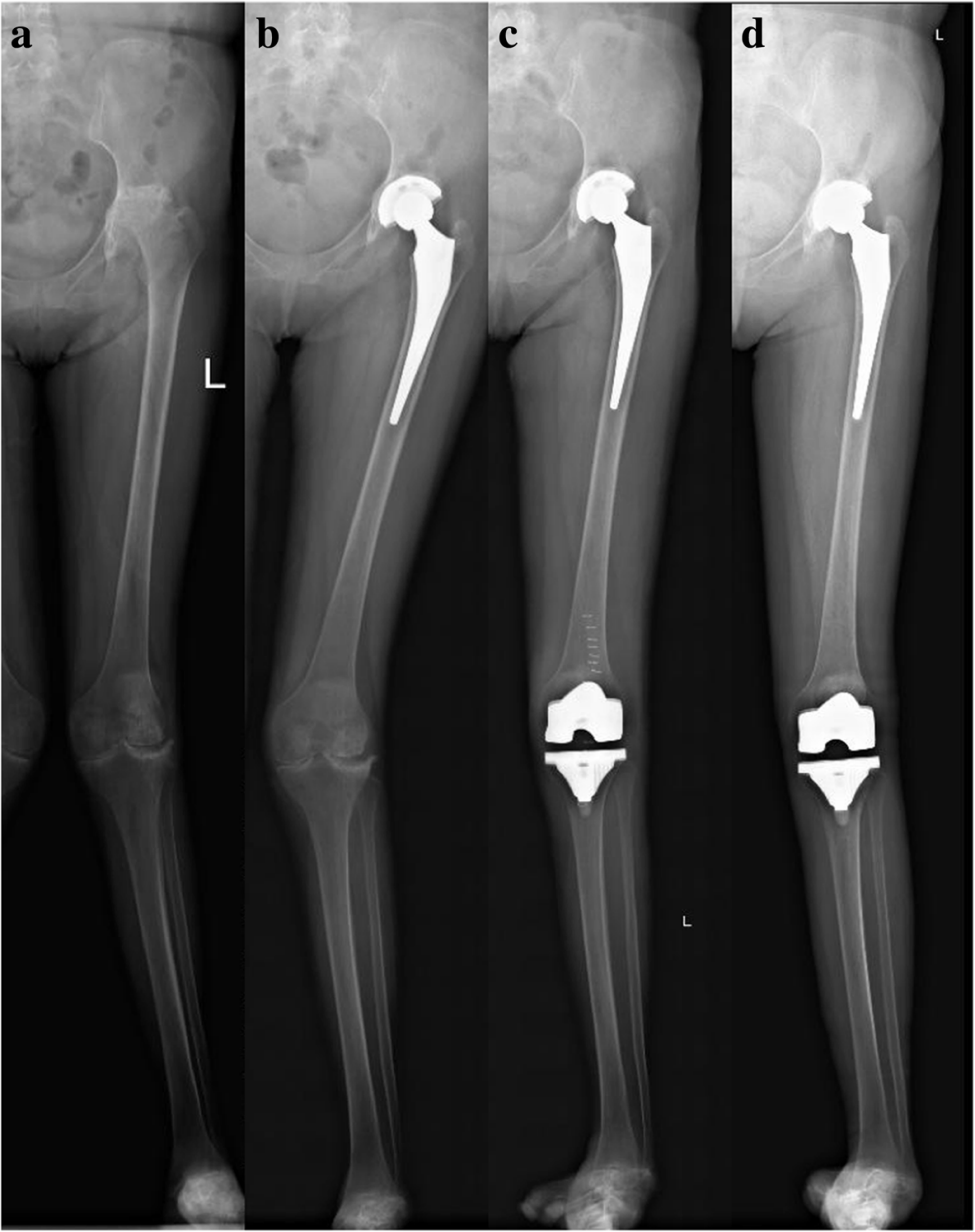
A 43-year-old female with rheumatoid arthritis receiving TKA subsequent to ipsilateral THA. X-ray radiography at preoperative (**a**) and radiography at 3-month follow-up after THA (**b**). Seven months after THA, the patient received ipsilateral TKA. The postoperative radiograph of the lower extremity (**c**) showed stable implant fixation. Postoperative radiographic image (**d**) at 7-year follow-up demonstrated that the acetabular, femoral, and tibial components were stable

**Fig. 3:**
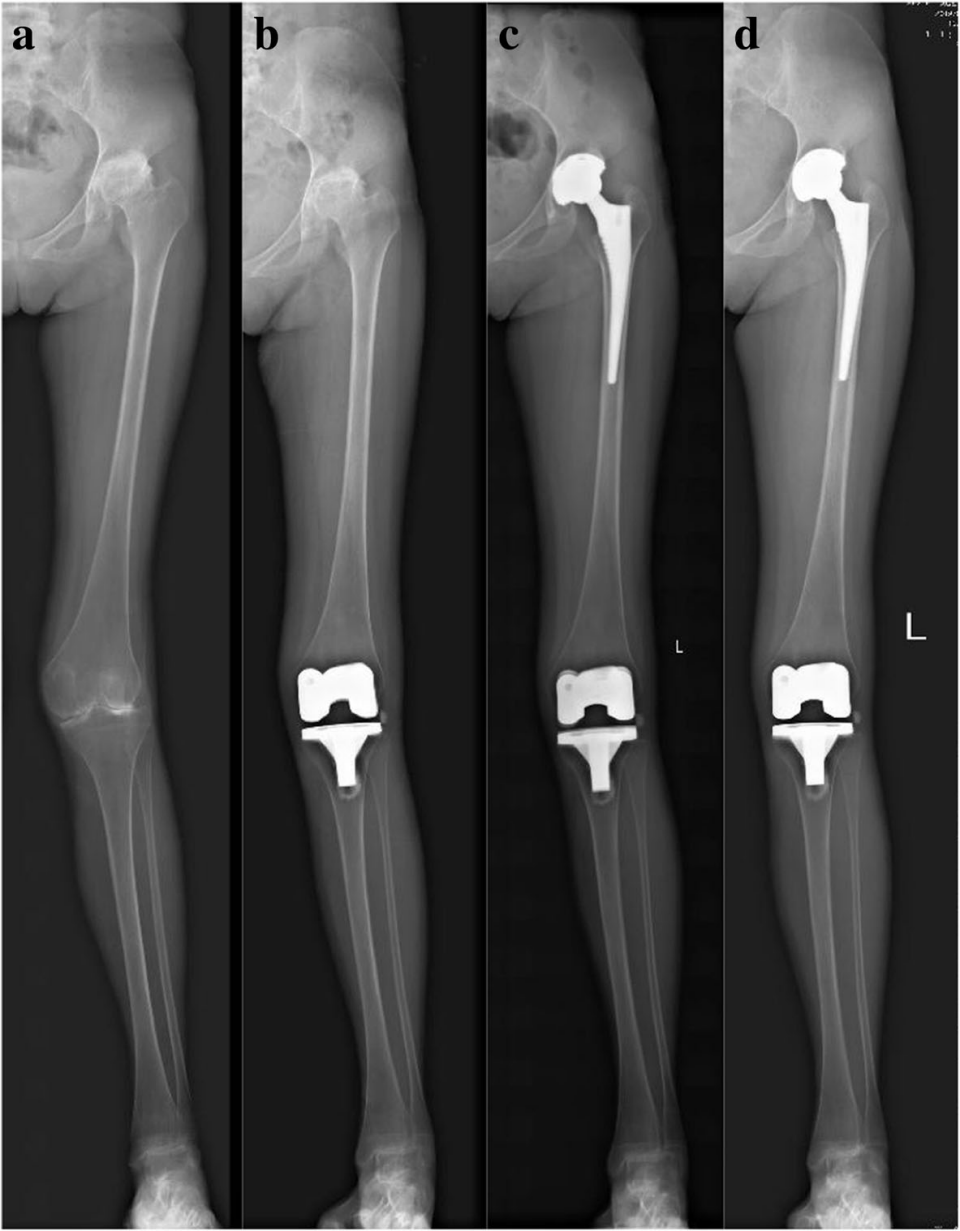
Radiographs (**a**-**d**) of a 27-y-old woman who underwent THA subsequent to ipsilateral TKA for rheumatoid arthritis. **a** Preoperative lower extremity image. **b** Radiograph of the patient undergoing TKA at 6-month follow-up. One year after prior TKA, the patient underwent ipsilateral THA. **c** The radiograph of the lower extremity at 6-month follow-up after THA showed stable implant fixation. **d** Postoperative radiograph of acetabular, femoral, and tibial components at 8-year follow-up showed stable implant fixation

There was also no significant difference in preoperative radiographic measurements between the 2 groups, including HKA, KAA, FO, and LLD (Table 2). However, preoperative mean HKA and FTA indicated valgus alignment in both groups (184.1° vs 184.1°, 173.0° vs 173.2°, *p* > .05). The comparisons of lower limb alignment parameters were based on the measured data of the full-length weight-bearing radiographs, as shown in Figs. 2 and 3. Relative to the mechanical and anatomical alignment, HKA and FTA were corrected to neutral after surgery (*p* < .001). The postoperative FO, LLD, and cup inclination did not differ between the 2 groups (*p* = .967, .137, .323).

**Fig. 4:**
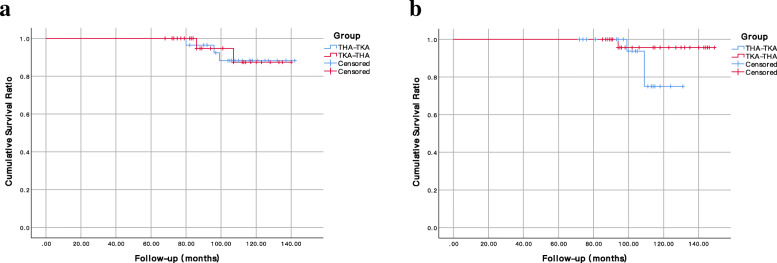
Kaplan-Meier survival curve with revision of THA (**a**) and TKA (**b**) for any reason other than infection as the endpoint

No periprosthetic infection or deep venous thrombosis was identified during the follow-up in each group (Table 3). There was 1 case of sciatic nerve palsy patient in the TKA-THA group after THA who recovered spontaneously within 9 months without residual symptoms. Intraoperative fracture occurred in 4 hips (1 in the distal femur and 3 in the proximal femur) and 1 knee without displaced cracks or perforation; all the fractures were treated with immediate cerclage wire fixation. Two patients (2 hips) experienced postoperative dislocation in the THA-TKA group who were treated with closed manipulative reduction and confined to bed for 4 weeks. Two patients in the THA-TKA group and 1 patient in the TKA-THA group had an over 5 mm subsidence of the femoral stem and underwent stem revisions with subsequent stabilization and evidence of fibrous stability at the final follow-up. Of the patients with aseptic revisions, in the THA-TKA group, two femoral revisions were performed for stem subsidence, and one acetabular revision was performed for recurrent dislocation. For ipsilateral TKA, two knee revisions were performed for tibial aseptic loosening, and 1 was performed for instability. In contrast, in the TKA-THA group, one femoral stem revision was performed for subsidence, one modular liner was changed because of dissociation of the highly cross-linked polyethylene insert from the outer shell, and one knee revision was performed due to tibial implant loosening. There was no difference between the THA-TKA group and the TKA-THA group with respect to the complication rate and overall revisions (*p* > .05). Considering aseptic revision as an endpoint (Fig. 4), THA survival at 8 years was 94.7% (95% confidence interval 84.7–99.9%) and 92.4% (95% confidence interval 87.4–97.4%) in the THA-TKA group and TKA-THA group, respectively (log-rank, *p* = .939). In contrast, the TKA survival at 8 years was 95.7% (95% confidence interval 87.3–99.9%) and 100.0% in the THA-TKA group and TKA-THA group, respectively (log-rank, *p* = .187).

**Table 3:** Complications and revisions

Complications	THA-TKA group	TKA-THA group	*P* value
	*n* = 28	*n* = 28	
Postoperative PFF	4	2	0.669
Early postoperative dislocation	2	0	0.154
Femoral component stability			0.429
Bony stable	22	25	
Fibrous stable	5	3	
Unstable	1	0	
Femoral stem subsidence	2	1	1.000
Revision			
Total hip revision	3	2	0.939
Total knee revision	3	1	0.187

*PFF* Postoperative periprosthetic fracture

## Discussion

Previous studies demonstrated that biomechanical changes and loading redistribution after prior THA or TKA may affect the ipsilateral hip or knee [5–9]. However, no study has compared the clinical outcomes and implant survivorships in patients who underwent different sequences of ipsilateral THA and TKA. The most important finding of the present study was that there was no significant difference between the 2 groups in HHS, KSS, and SF-12 score at the final follow-up; confirming our hypothesis.

Only one study [12] assessed the influence of a prior THA on axial alignment and the clinical outcome of a subsequent ipsilateral TKA by comparing patients who underwent TKA followed by ipsilateral THA and those who only underwent TKA. Compared with the most comparable study by Asensio-Pascual et al. [12], the clinical scores from our results were lower than those reported in their study, with postoperative HHS, KSS-knee, and KSS-function scores of averaged 86.4, 87.6, 88.3, respectively in the THA-TKA group. A possible interpretation is that disease of our study, including RA, which is involved in multiple joint sites, including metatarsal phalangeal joints and the ankle, might be present even after TJA. The immobility of other lower limb joints could lead to difficulty ambulating or ascending stairs despite evident improvements in hip and knee function. If we compared the clinical scores in OA patients from our study with the results of Asensio-Pascual et al. [12], the mean postoperative HHS, KSS-knee, and KSS-function score was 82.6, 86.0, 85.0 in the THA-TKA group, respectively, which demonstrated similar results.

Comparison of the radiological results and complication rate with the only study [12] is difficult because it evaluated lower limb alignment without full-length weight-bearing radiographs and did not systematically report the results of complications. Patients undergoing ipsilateral hip and knee surgeries presented preoperative valgus deformity in the current study. Preoperative knee valgus deformity was associated with advanced RA, which was in agreement with a previous study [26]. Preoperative valgus deformity means that the procedure of TKA is much more technically challenging, including obtaining a proper component rotational alignment and balancing soft tissue in both flexion and extension with the least constraint. Although knees with preoperative valgus were corrected to neutral and no significant differences were detected between the 2 groups in axial alignment at the last follow-up, our overall implant survivorship of TKA in the THA-TKA group was lower than that reported in Asensio-Pascual et al. [12], with 96.6% survivorship at 7.2 years. A possible explanation could be that aseptic revision after TJA seems to be higher in patients with RA than tin hose with OA. Previous studies reported consistent outcomes to ours: a Dutch study [27] reported implant survival rates of 58% in RA patients, whereas the survival rate was 95% in OA patients in 18-year follow-up; Feng et al. [28] reported implant survival rates of 78.3% in RA patients compared to 92.7% in OA patients in 15-year follow-up; A meta-analysis study [29] reported that aseptic revision rates of 7.7% (46/594) in RA patients compared to 5.7% (52/904) in OA patients.

In clinical practice, if the ipsilateral hip and knee simultaneously meet the indications for TJA, most surgeons would suggest performing THA before TKA. Active flexion and extension of the knee depend largely on free hip function, and knee pain is always associated with ipsilateral hip dysfunction. However, certain affected multiple joint diseases, such as RA, normally erode the knees first, leading to compulsory TKA. Then, the disease severity of the ipsilateral hip gradually developed to a degree that met the indication for THA. We agreed with the idea that the sequence of arthroplasties should depend on the severity of symptoms and that the most symptomatic joint of the hip or knee should be replaced first [8, 30].

Although biomechanical changes and redistribution of loading of the lower limb were detected after THA or TKA in certain studies [5–9], the influence of a prior TJA on the subsequent ipsilateral THA or TKA is limited. Consistent with the findings of the current study, Foucher et al. [10] reported no increase in biomechanical loading during gait on the ipsilateral knee after THA. Likewise, other studies demonstrated that the changes in the axial alignment of the lower extremity after THA could result in an increased overload on the contralateral knee rather than ipsilateral knee, which was characterized by compensation to minimize the loading of the affected limb [7]. Thus, patients who undergo THA may have higher risks of developing OA in the contralateral knee than in the ipsilateral knee [31].

To date, this is the first study to compare the mid- to long-term results of patients who underwent different sequences of ipsilateral THA and TKA. The strengths of this study include the completeness of clinical and radiographic data, uncemented hip prostheses and posterior-stabilized knee prostheses, and the homogeneity of the surgical technique. However, we note that there are several limitations as well. Firstly, the retrospective design decreased the level of evidence and implied a selection and recall bias. Secondly, the small sample for each group may be insufficient power to detect difference in clinical and radiological outcomes. However, it was the largest sample-size study focused on the influence of different sequences of ipsilateral THA and TKA. Prospective data retrieval studies with larger samples are needed. Furthermore, we also recognize that our outcomes may suffer from bias due to different indications of the 2 diseases and different implants used in hip or knee arthroplasty. To minimize the possible bias of hip implants, case-control matching was performed for protheses of THA. Finally, only coronal alignment was evaluated in the present study, although the rotational alignment and patellar tilt are also important for the success of TKA. However, this study focused on modification of the mechanical axis, which is mainly present in coronal alignment. Future research with three-dimensional CT would be useful in studying modifications in rotational alignment.

## Conclusion

Patients undergoing different ipsilateral THA and TKA sequences could achieve similar favorable clinical outcomes. Total joint arthroplasty can be performed safely with excellent outcomes in patients with a history of prior ipsilateral THA or TKA.

## Data Availability

The datasets used or analyzed in the current study are available from the corresponding author up reasonable request.

## Abbreviations

TJA: Total joint arthroplasty
THA: Total hip arthroplasty
TKA: Total knee arthroplasty
RA: Rheumatoid arthritis
OA: Osteoarthritis
HKA: Hip-knee-angle
ITB: Iliotibial band
LCL: Collateral ligament
POP: Popliteus tendon
HHS: Harris Hip Score
KSS: Knee Society Score
SF-12: Short Form-12 Score
FTA: Femorotibial angle
FO: Femoral offset
LLD: Limb length discrepancy
PFF: Periprosthetic fracture
PCS: Physical component summary
MCS: Mental component summary
